# The roles of carboxylesterase and CYP isozymes on the *in vitro* metabolism of T-2 toxin

**DOI:** 10.1186/s40779-015-0041-6

**Published:** 2015-07-02

**Authors:** Ni-Ni Lin, Jia Chen, Bin Xu, Xia Wei, Lei Guo, Jian-Wei Xie

**Affiliations:** State Key Laboratory of Toxicology and Medical Countermeasures, and Laboratory of Toxicant Analysis, Institute of Pharmacology and Toxicology, Academy of Military Medical Sciences, Beijing, 100850 People’s Republic of China

**Keywords:** T-2 toxin, Cytochrome P450, Carboxylesterase, Metabolism, Human liver microsomes

## Abstract

**Background:**

T-2 toxin poses a great threat to human health because it has the highest toxicity of the currently known trichothecene mycotoxins. To understand the *in vivo* toxicity and transformation mechanism of T-2 toxin, we investigated the role of one kind of principal phase I drug-metabolizing enzymes (cytochrome P450 [CYP450] enzymes) on the metabolism of T-2 toxin, which are crucial to the metabolism of endogenous substances and xenobiotics. We also investigated carboxylesterase, which also plays an important role in the metabolism of toxic substances.

**Methods:**

A chemical inhibition method and a recombinant method were employed to investigate the metabolism of the T-2 toxin by the CYP450 enzymes, and a chemical inhibition method was used to study carboxylesterase metabolism. Samples incubated with human liver microsomes were analyzed by high performance liquid chromatography-triple quadrupole mass spectrometry (HPLC- QqQ MS) after a simple pretreatment.

**Results:**

In the presence of a carboxylesterase inhibitor, only 20 % T-2 toxin was metabolized. When CYP enzyme inhibitors and a carboxylesterase inhibitor were both present, only 3 % of the T-2 toxin was metabolized. The contributions of the CYP450 enzyme family to T-2 toxin metabolism followed the descending order CYP3A4, CYP2E1, CYP1A2, CYP2B6 or CYP2D6 or CYP2C19.

**Conclusion:**

Carboxylesterase and CYP450 enzymes are of great importance in T-2 toxin metabolism, in which carboxylesterase is predominant and CYP450 has a subordinate role. CYP3A4 is the principal member of the CYP450 enzyme family responsible for T-2 toxin metabolism. The primary metabolite produced by carboxylesterase is HT-2, and the main metabolite produced by CYP 3A4 is 3′-OH T-2. The different metabolites show different toxicities. Our results will provide useful data concerning the toxic mechanism, the safety evaluation, and the health risk assessment of T-2 toxin.

## Background

T-2 toxin (4β, 15-diacetoxy-8α-(3-methylbutyryloxy)-3α-hydroxy-12, 13-epoxytrichothece-9-ene) is a toxic secondary metabolite produced by various species of *Fusarium* growing on cereal grains [1, 2]. Because it has extensively contaminated crops and cereals worldwide, animals and humans have a high potential of intoxication from contaminated food and feed. Typical symptoms of intoxication induced by T-2 toxin are feed refusal, weight loss and vomiting, which are related to its inhibitory effects on protein, DNA and RNA synthesis, as well as immunosuppressive and cytotoxic effects [3, 4].

T-2 toxin is rapidly metabolized *in vivo*, for example, its half-life is only 21 ± 5 min after the intramuscular injection of 0.4 mg/kg to dogs [5]. Ten or more metabolites have been deduced [6] and the metabolic pathways for T-2 toxin include hydrolysis, hydroxylation, de-epoxidation, glucuronidation and acetylation [7].

Because of their important role in toxin metabolism, drug-metabolizing enzymes have attracted much attention in studies aimed at gaining an understanding of toxicity. The cytochrome P450 (CYP450) superfamily is the major phase I metabolic enzymes in the liver (the principal organ responsible for drug metabolism), and these enzymes participate in approximately 75 % of *in vivo* drug metabolism [8]. These enzymes are crucial for the metabolism of foreign chemicals, including drugs, carcinogens, pollutants, pesticides and herbal compounds, as well as endogenous substances, including steroids, fatty acids and cholesterol [9]. The metabolic effect of the CYP450 enzymes on T-2 toxin has also aroused recent interest. Meissonnier *et al.* [10] found reduced expression of CYP1A proteins and CYP1A-related activities (ethoxyresorufin O-deethylation and benzo-(a)-pyrene hydroxylation) in pigs after the intake of feed contaminated with T-2 toxin. Osselaere *et al*. [11] demonstrated significant down-regulation of CYP1A4, CYP1A5 and CYP3A37 mRNA expression when broilers were fed a diet contaminated with T-2 toxin.

Carboxylesterase is another important type of phase I enzyme that metabolizes toxic substances [12, 13]. Johnsen *et al*. [14] found that T-2 toxin was rapidly hydrolyzed into the main metabolite HT-2 toxin by carboxylesterase in a rat liver microsomal fraction.

However, the effects of phase I drug-metabolizing CYP450 and carboxylesterase enzymes in the metabolism of T-2 toxin in humans have yet to be demonstrated. To address this issue in this study, human liver microsomes (HLMs) were used to investigate the metabolism of T-2 toxin by CYP450 enzymes and carboxylesterase employing a chemical inhibition and a recombinant method. We believe the results can provide information about the mechanism of toxicity of the T-2 toxin and other structurally similar toxins.

## Methods

### Chemicals and reagents

T-2 toxin, HT-2 toxin, neosolaniol (NEO), acetyl T-2, T-2 triol, T-2 tetraol and zearalanone (ZAN) with a purities greater than 98 %, bis (4-nitrophenyl) phosphate (BNPP), ethyleneglycol-bis (2-aminoethylether) -tetraacetic acid (EGTA), tetraisopropylpyrophosphamide (iso-OMPA), phenacetin, dextromethorphan, tolbutamide, α-naphthoflavone, sulfaphenazole, quinidine and tranylcypromine of analytical grade were purchased from Sigma Aldrich (St. Louis, MO, USA). We synthesized 3′-OH T-2 (purity of 99 %) in our laboratory. HPLC grade methanol and acetonitrile were obtained from DUKSAN (Sungkok, Korea). All other reagents were of analytical grade or better and purchased from Sino Pharm chemical reagent Co. Ltd (Beijing, China). Sterilized ultrapure water was generated with a Milli-Qultrapure water system (Millipore, Billerica, MA, USA). Human liver microsomes and nicotinamide adenine dinucleotide phosphate (NADPH) were purchased from Beijing Dingguo Changsheng Biotechnology Co., Ltd. (Beijing, China). CYP450 enzymes and S-mephenytoin were purchased from BD Gentest (Woburn, MA, USA). Midazolam and ketoconazole were purchased from the National Institutes for Food and Drug Control (NIFDC, Beijing, China).

All samples were separated in an Agilent 1200 HPLC system equipped with a binary pump, an Agilent control union, a degasser, and an auto sampler, and then detected by an Agilent 6430 triple quadrupole mass spectrometry (QqQ MS) equipped with an electrospray ion source (Agilent Co., California, USA). The introduction of the liquid phase was accomplished with an Agilent XDB C18 column (50 mm long, 4.6 mm diameter, 1.8 μm particle size, Agilent Co., California, USA) coupled with a Waters in-line filter kit (Waters Co., Milford, USA). All data were collected and analyzed by Agilent ChemStation software with MassHunter acquisition system (ver. B.04.00).

### Inhibitor phenotyping samples [15]

T-2 toxin (50 μL) at a concentration of 10 μmol/L was pre-incubated with 3 μl BNPP at a concentration of 3 mmol/L (an inhibitor of carboxylesterase), 1 μL EGTA at a concentration of 1 mmol/L (an inhibitor of paraoxonase), 5 μL iso-OMPA at a concentration 50 μmol/L (an inhibitor of acetylcholine esterase) with human liver microsomes at 37 °C for 5 min, respectively. HLM at a concentration of 0.5 mg/mL was then added to start the reaction, and 200 μL solution of 1 mmol/L ZAN in 1:3 methane: acetonitrile was added to stop the reaction after 30 min.

### Chemical inhibition method to determine the contribution of CYP450 enzymes [15]

The sample group was prepared as follows: 100 μL T-2 toxin at a concentration of 10 μmol/L was pre-incubated with 80 μL of α-naphthoflavone at a concentration of 10 μmol/L (an inhibitor of CYP1A2), sulfaphenazole at a concentration of 40 μmol/L (an inhibitor of CYP2C9), tranylcypromine at a concentration of 400 μmol/L (an inhibitor of CYP2C19), quinidine at a concentration of 10 μmol/L (an inhibitor of CYP2D6), or ketoconazole at a concentration of 10 μmol/L (an inhibitor of CYP3A4), respectively, in HLMs at 37 °C for 5 min. NADPH (20 μl) at a concentration of 1 mmol/L was then added to start the reaction, and a solution of 1 mmol/L ZAN in 1:3 methane: acetonitrile was added to stop the reaction after 30 min.

The inhibitor group was prepared as follows: all procedures were same as for the sample group, except that the carboxylesterase inhibitor BNPP was added at the beginning. For the positive control group, all procedures were same as the sample group, except that no isozyme inhibitor was added. For the negative control group, all procedures were same as the sample group, except that no inhibitor was added.

Each sample was prepared in triplicate. After the reactions were terminated, all samples were vortexed for 2 min and then centrifuged at 14,000 r/min for 10 min. The supernatant was injected in the HPLC-QqQ MS.

### Recombinant CYPs test [15]

All the procedures were same as for the sample group, except that the CYP450 isozyme was used instead of the liver microsomes. The isozymes included CYP2C19, CYP3A4, CYP2C9, CYP2A6, CYP2B6, CYP2D6, CYP1A2, CYP2C8 and CYP2E1, at a concentration of 25 nmol/L.

To determine the effects of NADPH, we used the same incubation conditions as for the sample group. Moreover, we added the stop solution at 0, 1, 2, 3, 4, 5, 10, 15, 20, 30 min to stop the reaction.

### Statistical analysis

The data were expressed as the mean ± SD and analyzed by an ANOVA test for independent samples using the SPSS 11.5 statistical software. The level of significance was *P* < 0.05.

### HPLC-QqQ MS parameters

The analysis was followed our previously established method with slight modifications [16]. The separation was performed on an Agilent XDB C18 column (50 mm long, 4.6 mm diameter, 1.8 μm particle size) coupled with a Waters in-line filter kit. The mobile phase A was 5 mmol/L ammonium acetate in water, and the mobile phase B was 5 mmol/L ammonium acetate in methanol. The gradient profile was 65 % B held from 0 to 0.5 min, followed by a linear increase to 100 % B over 3 min that was held for 2.5 min, then a decrease to 65 % B over 0.1 min that was held for 0.9 min, for a total run time of 7.0 min. The flow rate was 0.6 mL/min, the injection volume was 5 μl, and the column temperature was set to 50 °C.

The HPLC eluent was transferred by the XDB-C18 chromatographic column after the guard column to an Agilent triple-quadrupole mass spectrometer (QqQ MS) in the positive electrospray ionization mode. The capillary voltage was set to 3500 V, the source temperature was set at 350 °C, and the drying gas was set at 9 L/min. All of the analytes were measured in the multiple reaction monitoring (MRM) mode. All other parameters listed in Table 1.

**Table 1:** Parameters for the analytes and internal standard in HPLC-QqQ MS

Analytes	Ion pairs (m/z)	Fragmentor (V)	Collision (V)
NEO	400.1/185.0	85	15
3'-OH T-2	500.2/185.0	100	20
Triol	383.0/215.0	90	2
HT-2	442.2/215.0	95	8
T-2	484.2/185.0	110	15
ZAN (IS)	321.2/190.1	80	18
Acetyl T-2	526.2/287.1	105	10

## Results and discussion

To investigate the contribution of the CYP450 subfamily and carboxylesterase on the metabolism of T-2 toxin, we used chemical inhibition and recombinant methods. However, besides measuring changes in the T-2 toxin itself, we also simultaneously measured six of its principal metabolites to determine their fluctuation.

### Method evaluation

First, we established an HPLC-QqQ MS method for measuring T-2 toxin and its six metabolites in HLMs with ZAN as the internal standard. Good linearity (*R*^2^ > 0.990) in a range from 0.005 to 20.0 μmol/L was obtained, the limit of detection was between 0.001 and 0.067 μmol/L, and the recovery varied from 82.2 % to 119.1 % with relative standard deviations of 6.5 % to 14.9 %. The method was proved to be rapid, reproducible, and sensitive and was successfully applied to the metabolic research of T-2 toxin.

### Contribution of different enzymes

To include most types of phase I drug-metabolizing enzymes existing in HLMs, we investigated the CYP450 enzymes, carboxylesterase, paraoxonase and acetylcholine esterase*.* We first examined the self-degradation of T-2 toxin and found that T-2 toxin was stable in phosphate buffer for over one hour. We then added NADPH to activate the CYP450 enzymes, or did not add NADPH to observe the effects of other enzymes on T-2 toxin metabolism.

**Fig. 1:**
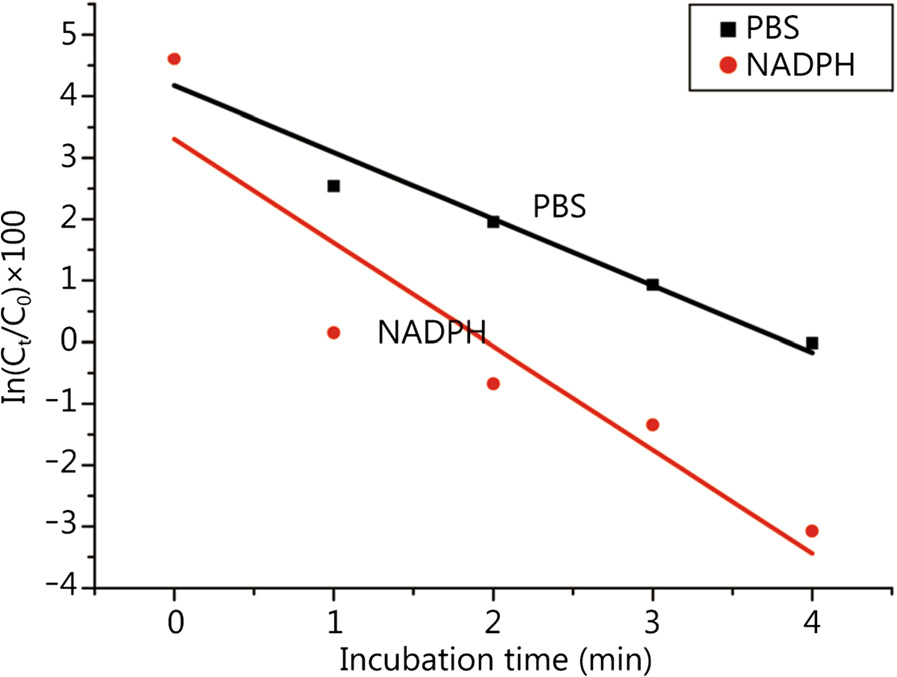
Semi-logarithm plot of the remaining percentage of the T-2 toxin in HLMs *vs* incubation time

The results are shown in Fig. 1. A common single exponential decay model used in metabolic stability studies [17] with the formula of t_1/2_ = −0.693/ln[(C_t_/C_0_) × 100] was plotted in Fig. 1, where t is the reaction period, C_t_ is the concentration of the parent compound at time t, and C_0_ is the initial concentration in the incubation system. Figure 1 shows that T-2 toxin is rapidly depleted if the CYP450 enzymes are unactivated, with a t_1/2_ (NADPH) of 0.4 min and t_1/2_ (PBS) of 0.6 min. These data clearly suggest that other types of enzymes have a greater contribution to T-2 toxin metabolism than the CYP450 enzymes.

**Fig. 2:**
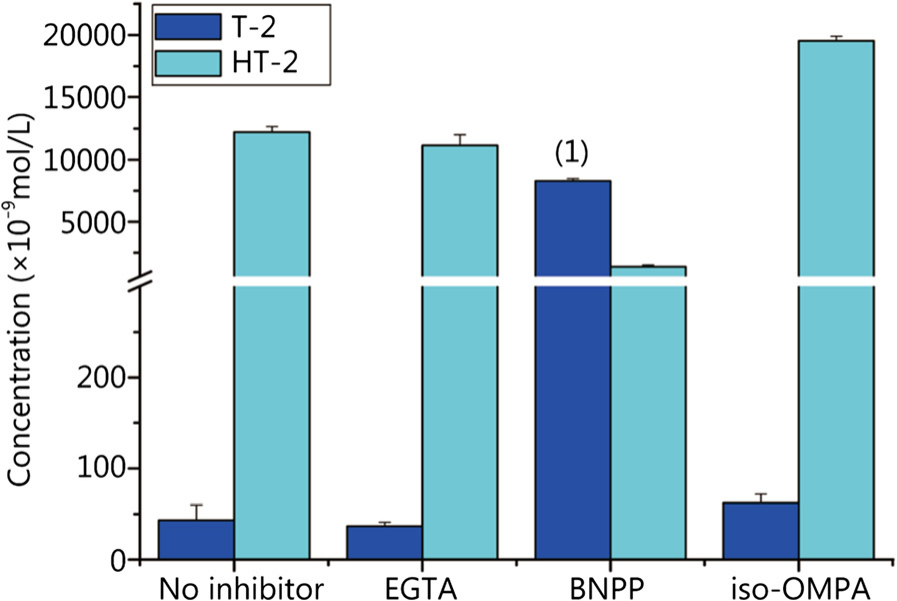
Influence of different enzyme inhibitors on T-2 toxin metabolism. (1) *P* < 0.05 compared with no inhibitor group

The precise contribution of each type of enzyme other than CYP450 was determined with a corresponding chemical inhibitor. The carboxylesterase inhibitor BNPP, the paraoxonase inhibitor EGTA and the acetylcholine esterase inhibitor iso-OMPA were added to the incubation system with T-2 toxin. The results are shown in Fig. 2, which employs the concentration relationship between the T-2 toxin and its primary metabolite HT-2 to illustrate the effect of each type of enzyme. The initial concentration of T-2 toxin was 10 μmol/L. More than 80 % T-2 toxin was metabolized when EGTA, iso-OMPA or no inhibitor was added, but approximately 80 % of the T-2 toxin remained unmetabolized when BNPP was added. These results demonstrate that EGTA and iso-OMPA have little influence on T-2 toxin metabolism, but BNPP greatly affects T-2 toxin metabolism. We concluded that carboxylesterase was the predominant enzyme for T-2 toxin metabolism. The results confirmed the importance of carboxylesterase in the detoxification of trichothecenes as Johnsen *et al*. [14] concluded. However, Johnsen *et al*. used rat liver but we used human liver, so our experiment was more reflective of human metabolism.

### CYP450 enzyme

**Fig. 3:**
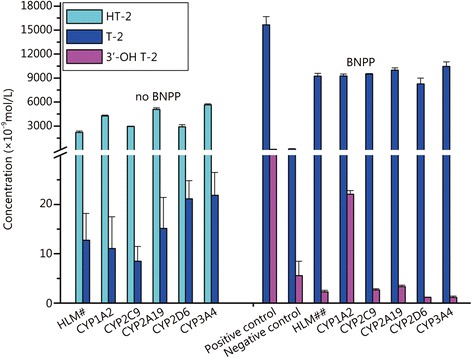
Influence of CYP450 inhibitors on T-2 toxin metabolism. HLM# is T-2 toxin incubated with the HLMs as same as in the sample group except that all the isozyme inhibitors were added; and HLM## is T-2 toxin incubated with the HLMs the same as in the sample group with BNPP except that all the inhibitors were added

CYP1A2, CYP2C9, CYP2C19, CYP2D6 and CYP3A4 play the most important roles in the CYP450 subfamily, and they were recommended by the United States Food and Drug Administration for the evaluation of drug metabolism in humans [18, 19]. In this study, we employed two methods, a chemical inhibitor method and a recombinant method with a normalization approach, to evaluate the effects of the CYP450 isozymes. For the chemical inhibitor method, we chose five different chemical inhibitors of the five important isozymes and CYP2B6. Table 2 shows that, in the presence of the carboxylesterase inhibitor BNPP, approximately 3 % T-2 toxin was metabolized when a CYP450 enzyme inhibitor was added. We also observed the same trend in the concentrations of T-2 toxin and its principal metabolites. As shown in Fig. 3, almost all of the T-2 toxin is metabolized without the CYP450 enzyme inhibitor and BNPP, while T-2 toxin remains in the presence of the CYP450 enzyme inhibitor and BNPP; in the latter situation, CYP3A4 shows the highest metabolism ability to T-2 toxin of all six CYP450 isozymes. The major metabolite in the absence of BNPP was HT-2 and 3′-OH T-2 was produced in the presence of BNPP.

**Table 2:** Different inhibition effects of CYP450 isozymes on T-2 toxin metabolism in HLMs

Isozyme	Inhibitor	Percent of remaining T-2 toxin without BNPP (%)	Percent of remaining T-2 toxin with BNPP (%)
CYP2C19	Trans-2-phenylcyclopropylamine	0.19 ± 0.08	107.24 ± 3.16
CYP3A4	Ketonazle	0.27 ± 0.06	112 ± 6.10
CYP 2C9	Sulfaphenazole	0.10 ± 0.04	102.10 ± 0.80
CYP 2D6	Quinidine	0.26 ± 0.06	96.87 ± 7.87
CYP 1A2	α-naphthol	0.14 ± 0.08	99.37 ± 2.68
Negative control	No inhibitor	0.16 ± 0.07	97.10 ± 8.34
Control^a^	No inhibitor	100 ± 3.86	100 ± 3.86

^a^The termination solution was added at the beginning in HLMs and no NADPH added to active the reaction

We also used a recombinant method for the CYP450 subfamily only, by which we could calculate the contribution of each isozyme to T-2 metabolism by using the metabolic velocity value for the T-2 toxin and the concentration of each CYP isozyme (Ccyp) [20], and the total normalized rate (TNR) [21]. The metabolic velocity was calculated by the formula Vm = (C_0_- C_t_)/t × Ccyp (protein), where Vm is the metabolic velocity, t is the reaction period, C_t_ is the concentration of the parent compound at time t, and C_0_ is the initial concentration in the incubation system where Ccyp was the content of specific CYP450 in the human liver.

TNR was given by the formula: Percent TNR = (NR/TNR) × 100 %.

The normalized rate (NR) was obtained by multiplying the reaction rate for each isoform by the specific CYP450 content in the HLMs. The NRs were then summed to give the TNR.

Table 3 shows the results, which followed the descending order of CYP3A4, CYP2E1, CYP1A2, CYP2C9, CYP2B6 or CYP2D6 or CYP2C19, in which CYP3A4 contributed the most to T-2 toxin metabolism, a value close to 90 %.

**Table 3:** Contribution of CYP450 isozyme to T-2 toxin hepatic metabolism assessed by the TNR method

CYP isozyme type	CYP2C19	CYP 3A4	CYP2C9	CYP2B6	CYP2D6	CYP1A2	CYP2E1
Vm (nmol/T-2/min nmol protein)	0.86 ± 0.02	13.98 ± 3.46	0.47 ± 0.03	1.2 ± .02	1.73 ± 0.03	1.37 ± 0.05	1.41 ± 0.07
Percentage of total activity	0.63	89.70	1.72	0.69	0.63	3.01	3.62

The two methods yielded orders of contribution to T-2 toxin metabolism that were slightly different, but both methods indicated that the CYP3A4 isozyme was the major contributor. Considering that the chemical inhibitor was nonspecific in some cases, meaning that two or more types of enzymes could be inhibited, the recombinant method probably reflects more reliable results.

**Fig. 4:**
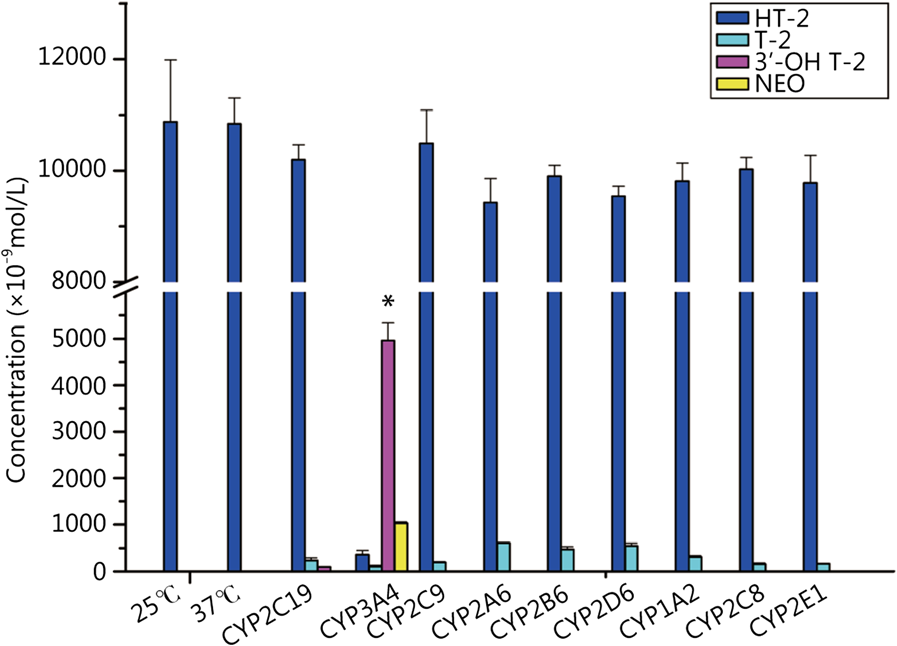
The type and concentration of different T-2 toxin metabolites with different phenotypes of CYP450 recombinants. * *P* < 0.05 compared with 37 °C group. The order of T-2 toxin metabolism was CYP3A4, CYP2D6, CYP2C19, CYP1A2, CYP2C9 at 25 or 37 °C. The termination solution was added at the beginning when T-2 toxin was incubated at 25 or 37 °C

Wang *et al*. [22] determined that porcine CYP3A46 mainly converted T-2 toxin to 3'OH T-2, which was further confirmed using purified CYP3A46 protein. They added ketoconazole, which is a potent inhibitor of CYP3A4-mediated metabolism in humans that has been widely used as a probe for CYP3A inhibition in other animal species [23]. As the dosage of ketoconazole increased, the amount of 3'OH T-2 decreased. Recombinant pig CYP3A22 converted T-2 toxin into 3′-OH T-2 toxin and HT-2 toxin into 3′-OH HT-2 toxin *in vitro* [24]. Pig CYP3A22 eliminated T-2 and HT-2 toxins primarily by 3′-hydroxylation of the isovaleryl groups. It was suggested that CYP3A22 was critical for xenobiotic metabolism and the endogenous biochemical biotransformation of trichothecene mycotoxin in pigs. CYP1A5 played an important role in chickens by hydroxylating T-2 toxin to 3′-OH T-2 [25]. CYP3A37 converted T-2 toxin to 3′-OH T-2, and the T-2 hydroxylation activity of CYP3A37 was strongly inhibited by ketoconazole. Chicken CYP3A37 can also catalyze erythromycin N-demethylation, which is another CYP3A-specific activity. These findings indicate that chicken CYP3A37 may have a broad substrate spectrum similar to its human homologue CYP3A4 [26]. The metabolism of T-2 toxin has been extensively studied in animals; however, data in HLM and human recombinant enzymes are sparse. Our results showed CYP3A4 played major roles in recombinant enzymes in human liver microsomes, and the 3′-OH T-2 toxin was the corresponding product (Fig. 4). The results were similar to those in pigs and chickens in which CYP3As-specific activity had similar effects and the same product was produced. Chicken CYP1A5 was involved in the metabolism of T-2, but the type of CYPs was different in human because of the species difference.

## Conclusion

From systematic research on carboxylesterase and CYP450 enzymes for T-2 toxin metabolism in HLMs, we concluded that the carboxylesterase plays the primary role and CYP450 enzymes are subordinate. CYP3A4 was the major isozyme in CYP450 subfamily that contributed to T-2 toxin metabolism. The major metabolite was HT-2 with carboxylesterase and 3′-OH T-2 with CYP3A4. Our results provide useful data about the toxic mechanism of T-2 toxin.

## Abbreviations

CYP: Cytochrome P450
HPLC-QqQ MS: High performance liquid chromatography-triple quadrupole mass spectrometry
DNA: Deoxyribonucleic acid
RNA: Ribonucleic acid
HLMs: Human liver microsomes
NEO: Neosolaniol
ZAN: Zearalanone
BNPP: Bis (4-nitrophenyl) phosphate
EGTA: Ethyleneglycol-bis (2-aminoethylether) -tetraacetic acid
iso-OMPA: Tetraisopropylpyrophosphamide
NADPH: Nicotinamide adenine dinucleotide phosphate
NIFDC: National Institutes for Food and Drug Control
PBS: Phosphate buffered solution
